# New Insights Into the Biosynthesis of Typical Bioactive Components in the Traditional Chinese Medicinal Fungus *Cordyceps militaris*

**DOI:** 10.3389/fbioe.2021.801721

**Published:** 2021-12-17

**Authors:** Xiuyun Wu, Tao Wu, Ailin Huang, Yuanyuan Shen, Xuanyu Zhang, Wenjun Song, Suying Wang, Haihua Ruan

**Affiliations:** ^1^ Tianjin Key Laboratory of Food Science and Biotechnology, College of Biotechnology and Food Science, Tianjin University of Commerce, Tianjin, China; ^2^ New College, University of Toronto, Toronto, ON, Canada

**Keywords:** biosynthesis, cordycepin, d-Mannitol, cordyceps polysaccharides, N^6^-(2-hydroxyethyl)-adenosine (HEA)

## Abstract

*Cordyceps militaris*, a traditional medicinal ingredient with a long history of application in China, is regarded as a high-value fungus due to its production of various bioactive ingredients with a wide range of pharmacological effects in clinical treatment. Several typical bioactive ingredients, such as cordycepin, D-mannitol, cordyceps polysaccharides, and N^6^-(2-hydroxyethyl)-adenosine (HEA), have received increasing attention due to their antitumor, antioxidant, antidiabetic, radioprotective, antiviral and immunomodulatory activities. Here, we systematically sorted out the latest research progress on the chemical characteristics, biosynthetic gene clusters and pathways of these four typical bioactive ingredients. This summary will lay a foundation for obtaining low-cost and high-quality bioactive ingredients in large amounts using microbial cell factories in the future.

## Introduction

*Cordyceps militaris* is an entomopathogenic filamentous fungus, belonging to division Ascomycota, class Sordariomycetes, order Hypocreales, family Clavicipitaceae. *C. militaris* has been demonstrated to be beneficial in the treatment of male reproductive problems (Chen et al., 2017), chronic kidney (Zhang et al., 2014; Hong et al., 2015), respiratory (Bai et al., 2020), heart, liver (Cheng et al., 2014; Liu et al., 2014; Fan et al., 2018) and lung diseases (Wang et al., 2016), hyperglycemia, hyperlipidemia, and cancer. Wild *C. militaris* is generally called Yong Chong Cao in China for its parasitizing within the insect host’s body in winter and forming grass-like fruiting bodies by absorbing nutrients from host’s body in summer. *C. militaris*, which is considered a model species of the genus, is acknowledged to produce many bioactive secondary metabolites, including cordycepin, D-mannitol, cordyceps polysaccharides, and HEA.

**FIGURE 1:**
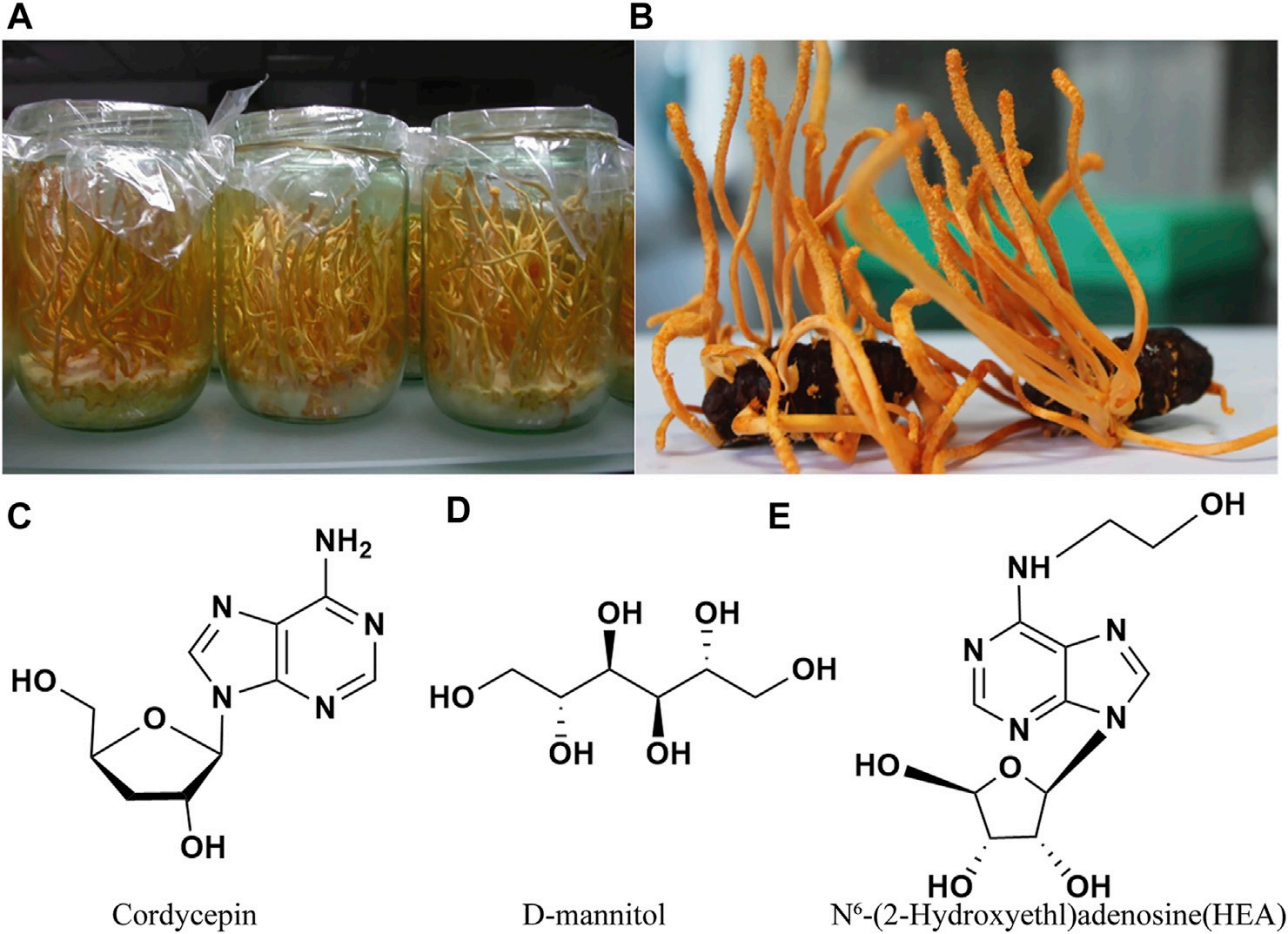
*Cordyceps militaris* cultured on a wheat medium **(A)** and on pupae **(B)** (Guo et al., 2016), and three typical bioactive components **(C–E)** of *Cordyceps militaris*.

The most direct way to obtain these four bioactive secondary metabolites is to separate and extract them from the wild and artificial cultivated *C. militaris* in the laboratory (Figure 1) (Tang et al., 2018; Li X. et al., 2019). However, the long growth cycle, harsh environmental requirements and other reasons, resulting in the technical cost of this kind of extraction industry may be relatively high, and it will also increase the burden on the industrial environment.

In recent years, the rise of synthetic biology has enabled many industrial microorganisms to produce various secondary metabolites to satisfy the demand of market (Teh et al., 2019). However, there are no systematical illustration about the synthesis pathways of these biologically active substances in *Cordyceps* spp. Therefore, we summarized the biosynthetic pathways of cordycepin, D-mannitol, cordyceps polysaccharides, and N^6^-(2-hydroxyethyl)-adenosine (HEA).

### Cordycepin

Cordycepin was first isolated in 1950 from liquid cultures of *C. militaris* (Cunningham et al., 1951). In terms of chemical structure, cordycepin is 3′-deoxyadenosine, consisting of two fused heterocycles and an imidazole as shown in Figure 1C. As a structural analog of adenosine and imidazole, cordycepin has been found to have a wide range of pharmacological activities, such as anti-tumor, immunomodulatory, anti-bacterial and anti-inflammatory effects (Nakamura et al., 2015; Li et al., 2017; Jin et al., 2018; Wang H. B. et al., 2019; Jang et al., 2019; Graeff et al., 2020; Liu et al., 2021).

Based on its industrial application potential in medicine, increasing studies in recent years have focused on the improvement of *C*. *militaris* strain for adequate and stable production of cordycepin, including genetic manipulations, genome shuffling, and mutagenesis and hybridization. However, the production of cordycepin remains challenging due to low product yields, high cost and complex extraction processes, limiting its industrial application. Given above, developing more efficient strategies for cordycepin production has been the focus of recent studies, among which the use of low-cost substrates and genetically engineered microbial strains might be the most promising approach. Therefore, an overview on cordycepin biosynthesis and related genetic engineering strategies with major points on the latest trends on study methods for the evolution of cordycepin biosynthesis and production is summarized in detail.

### *C. militaris* Strain Improvement

In general, genetic manipulations have been used to screen the cordycepin-producing strains. To our knowledge, strain improvement of *C. militaris* mainly includes three stages. Firstly, genome shuffling is used via a combination of traditional mutagenesis technology and cell fusion technology. It has been successfully applied to improve the production of cordycepin in *C. kyushuensis*, whereby the highest production of cordycepin reached 978.25 μg/g, representing a 9.63-fold increase over the parent strain (Wang et al., 2017). By contrast, the manipulation of individual genes mostly has only a limited effect which might be due to the lack of systematic regulation for cordycepin biosynthesis. For example, Lou *et al.* knocked out the *Cmfhp* gene of *C. militaris*, which was speculated to play a role in fruiting body development, nitric oxide (NO) metabolism, conidia formation, and also in cordycepin production. However, their results showed that the cordycepin yield of the strain did not increase significantly in the *Cmfhp* knockout strain (Lou et al., 2020). In addition, in a study of ribonucleotide reductase RNR (RNRL and RNRM) gene overexpression strains the content of cordycepin isolated from *C. militaris* transformants carrying RNRM was remarkably higher than that of the wild-type strain (Zhang et al., 2020b). Secondly, hybridization was performed for genetic recombination in single spores of *C. militaris*, the obtained strains after mutagenesis and hybridization could produce 6.63 mg/g cordycepin, which was increased by 35% than parent strain (Kang et al., 2017). Thirdly, a multifunctional plasma mutation system (MPMS) was applied to the improvement of strains, the results showed that the yield of cordycepin obtained from a MPMS treated strain named GYS60 reached to 7.88 mg/ml, which was more than 20 times higher than the yield of the wild-type strain (Zhang et al., 2020a).

At present, increased growth and cordycepin overproduction have been achieved using different *C. militaris* strains, but there are also many problems, such as the long growth cycle of the *C. militaris* and the instability of breeding strain, which hinder its application on an industrial scale. Thus, the construction of cell factories with a short growth cycle capable overproducing cordycepin has become a research hotspot.

### Biosynthesis of Cordycepin

**FIGURE 2:**
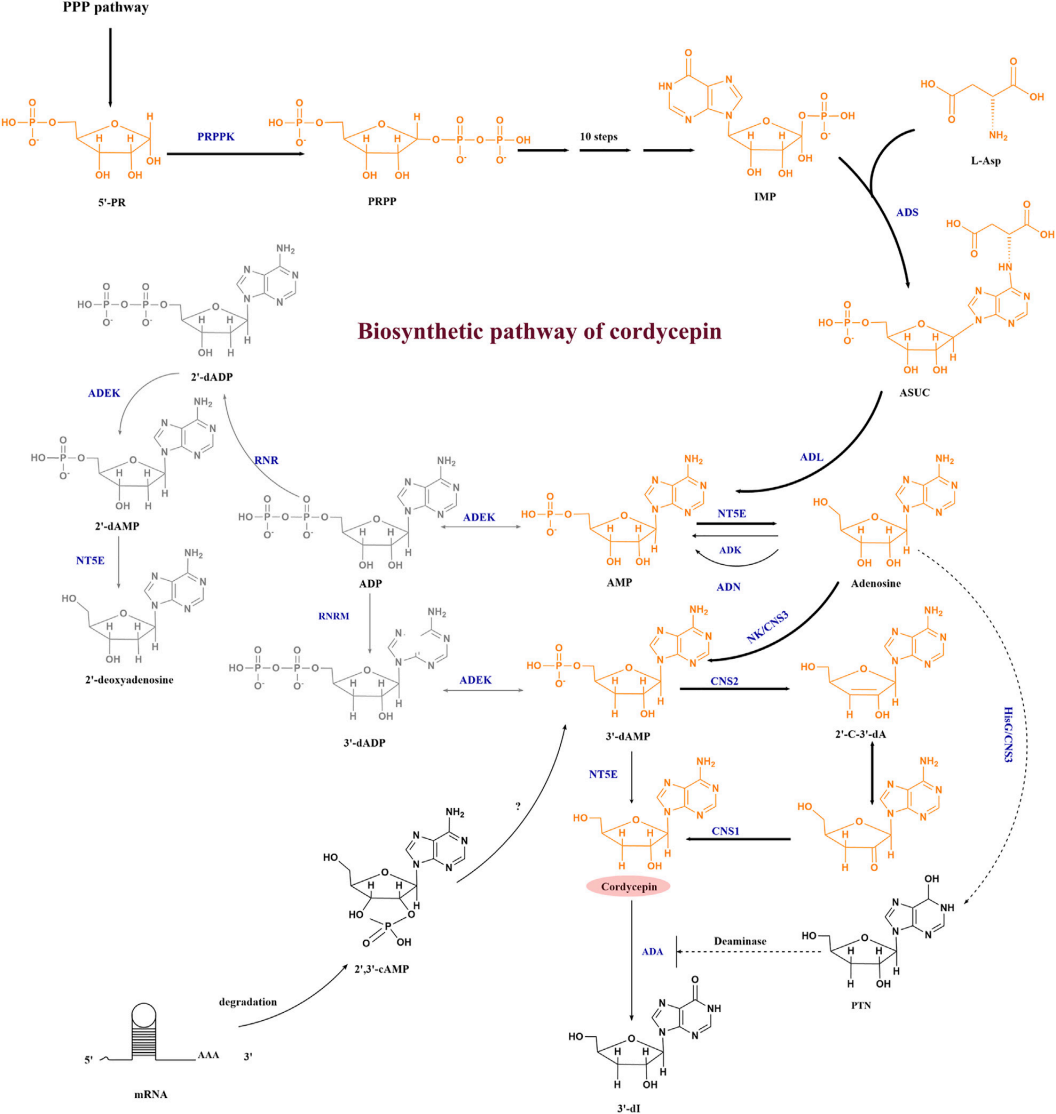
Cordycepin biosynthesis pathway. Abbreviations: 5′-RP, ribose-5-phosphate; PRPP, phosphoribosyl pyrophosphate; IMP, inosine monophosphate; ASUC, N6-(1,2-dicarboxyethyl)-AMP; AMP, adenosine-5′-monophosphate; ADP, adenosine diphosphate; 2′-dADP, 2′-deoxyadenosine diphosphate; 2′,3′-cAMP, 2′3′-cyclic monophosphate; 3′-dAMP, adenosine-3′-monophosphate; 2′-C-3′-dA, 2′-carbonyl-3′-deoxyadenosine; PTN, pentostatin; 3′-dI, 3′-deoxyinosine; PRPPK, phosphoribose pyrophosphate kinase; ADS, adenylosuccinate synthase; ADL, adenylosuccinate lyase; ADEK, adenylate kinase; NT5E, 5′-nucleotidase; ADK, adenosine kinase; ADN, adenosine nucleosidase; RNR, ribonucleotide reductases; RNRM, RNR small subunit, NK/CNS3, an N-terminal nucleoside kinase of Cns3; HisG/CNS3, a C-terminal HisG family of ATP phosphoribosyl transferases in Cns3; CNS2, HDc family of metal-dependent phosphohydrolase domain in Cns2; CNS1, the oxidoreductase/dehydrogenase domain in Cns1; ADA, adenosine deaminase. The bold arrow indicates the main synthesis direction of cordycepin, the dashed arrow represents the pathway that needs further verification, the full line represents the established pathway.

In order to obtain industrial cell factories that overproduce cordycepin, we need to explore the gene cluster needed for cordycepin synthesis and its synthesis pathway *in vivo*. Although cordycepin was discovered in *C. militaris* in 1950, the elucidation of its biosynthetic pathway has not been resolved for a long time due to the lack of powerful genome analyzing tools. Untill 2011, the adenosine metabolic pathways in *C. militaris* and the cordycepin biosynthesis genes were primarily disclosed based on genomics and transcriptomics analysis. The recent development of cordycepin biosynthesis pathways was summarized in Figure 2, which includes three pathways.

The first pathway of cordycepin biosynthesis in *C. militaris* is shown in yellow. The synthesis starts from PRPP (phosphoribosyl pyrophosphate) pathway, after which inosine monophosphate (IMP) and L-Asp are converted by adenylosuccinate synthase (ADS) to form N^6^-(1, 2-dicarboxyethyl)-AMP (ASUC). Then, AMP and adenosine are successively formed under the catalysis of adenylosuccinate synthase (ADL) and 5′-nucleotidase (NT5E). With the development of bioinformatics and omics technologies, four highly conserved protein coding genes named *cns1*-*cns4* were found to be related to the metabolism of adenosine in 2017 by comparing large amounts of orthologous proteins between *C. militaris* and *A. nidulans* with the efforts of Xia’s team (Xia et al., 2017). They proved that CNS1-CNS3 were responsible for cordycepin synthesis. The nucleoside/nucleotide kinase domain of CNS3 catalyzed the hydroxyl phosphorylation at the 3′-OH position of adenosine to form adenosine-3′-monophosphate (3′-AMP). At the same time, the C-terminal HisG domain of CNS3 was found to convert adenosine to pentostatin, which can inhibit the deamination reaction of cordycepin. The resulting 3′-AMP is then dephosphorylated to 2′-carbonyl-3′-deoxyadenosine (2′-C-3′-dA) by CNS2. Cordycepin is finally produced from 2′-C-3′-dA by oxidoreductase reactions mediated by CNS1 (Xia et al., 2017). In 2020, Zhao et al. (2019) identified a single gene cluster containing four genes encoding enzymes capable of synthesizing cordycepin and pentostatin simultaneously in *C. kyushuensis*, and named them *ck1*-*ck4*. This was consistent with the research of Xia et al. (2017).

The second pathway is shown in grey in Figure 2 in *O. sinensis*, the reduction of adenosine diphosphate (ADP) to 2′-deoxyadenosinediphosphate (3′-dADP) is catalyzed by highly conserved ribonucleotide reductases (RNR), and then adenylate kinase (ADEK) and 5′-nucleotidase (NT5E) are involved in phosphorylation and dephosphorylation in the adenosine metabolic pathway. RNR, ADEK, and NT5E were also revealed in the *C. militaris* genome in 2011 (Zheng et al., 2011), and both of *C. militaris* and *O. sinensis* have the capability of producing cordycepin. Thus, according to the similarity to 2′-deoxyadenosine biosynthesis, Xiang *et al.* predicted that the biosynthesis of cordycepin may proceed through a reductive mechanism after transcriptome analysis of the *O. sinensis* fruiting body (Xiang et al., 2014), but they didn’t verify the cordycepin biosynthesis pathway. Untill 2017, the recombinant *E. coli* which expressed CmRNR showed RNR activity on ADP but did not produce 3’ -deoxy ADP (Kato et al., 2017), which seemed to deny the participation of RNR in cordycepin biosynthesis in *C. militaris*. But in 2020, Zhang *et al.* indicated that the RNR consisted of two subunits: a large one (RNRL) and a small one (RNRM), RNRM could regulate the biosynthesis of cordycepin directly via the reduction of adenosine, as demonstrated by overexpressing RNRM in *C. militaris* (Zhang et al., 2020b).

The third pathway are branched from the precursor and the direct metabolite of 3′-dAMP. The 3′-AMP of cordycepin precursor can also be synthesized from 2′, 3′-cyclic AMP except from 3′-dADP and adenosine, which is degraded from mRNA degradation in *C. militaris* (Wongsa et al., 2020).

To validate whether some of these gene clusters mentioned above can enable other microbials to produce cordycepin, the *cns1*–*cns3* gene cluster were firstly expressed in *M. robertsii* (*Metarhizium robertsii*) (a closely related insect pathogenic fungus), and finally the recombinant *M. robertsii* produced cordycepin (Xia et al., 2017). Besides that, the budding yeast, *Saccharomyces cerevisiae* heterologous expressed either *cns1*–*cns3* or *cns1*–*cns2* could produce cordycepin (Xia et al., 2017; Huo et al., 2021). Therefore, the function of CNS3 in the synthesis of heterologous cordycepin has not yet been confirmed. At present, the specific functions of other related genes in the biosynthetic pathway of cordycepin have yet to be verified, and there are few studies on cordycepin cell factories, and a lot of related explorations are needed.

### D-Mannitol

In 1957, an active substance was first isolated from *C. sinensis* (Berkeley) Saccardo strain and initially named cordycepic acid (Cao et al.), and in 1963 its structure was revised to D-mannitol (Figure 1D). D-mannitol is a linear six-carbon polyol that can be considered an isomer of D-sorbitol, differing only in the orientation of the C2-OH group. On account of its unique physical and chemical characters, D-mannitol has valuable applications in medicine, fine chemicals, textiles, and food (Duan et al., 2018). It has been shown that D-mannitol can activate mitochondrial ATP-sensitive potassium (mK_ATP_) channels to protect heart (Feige et al., 2021). In addition, D-mannitol can also be a adjunctive therapy for acute promyelocytic leukaemia (Guo et al., 2021). Due to its wide range of applications, D-mannitol had a total market value of USD 209.4 million in 2015, and its demand seems to increase in the future (Wei et al., 2021). Therefore, many scientists have studied strategies to increase the biotechnological production of D-mannitol.

There are currently three main methods to produce D-mannitol, including chemical synthesis and biosynthesis. The chemical synthesis of D-mannitol starts from D-fructose, D-glucose, and D-mannose. And then after catalytic hydrogenation, the final product is obtained through stepwise crystallization (Jin and Li, 2006). However, the process of chemical synthesis requires large amounts of energy and produces problematic by-products, which severely limit its wide application (Cao et al., 2018). Besides that, there have many organisms in nature that can synthesize D-mannitol, such as higher plants, algae, lichens, bacteria and fungi. However, in photosynthetic organisms, D-mannitol is the main primary photosynthetic product and energy storage compound, its level is difficult to reach a large amount without affecting the growth of organisms. Therefore, more promising strategies have been exploited on lactic acid bacteria, filamentous fungi and *Escherichia coli* to produce D-mannitol via environmentally friendly biosynthetic pathways in recent decades.

### Synthesis of D-Mannitol in Lactic Acid Bacteria

**FIGURE 3:**
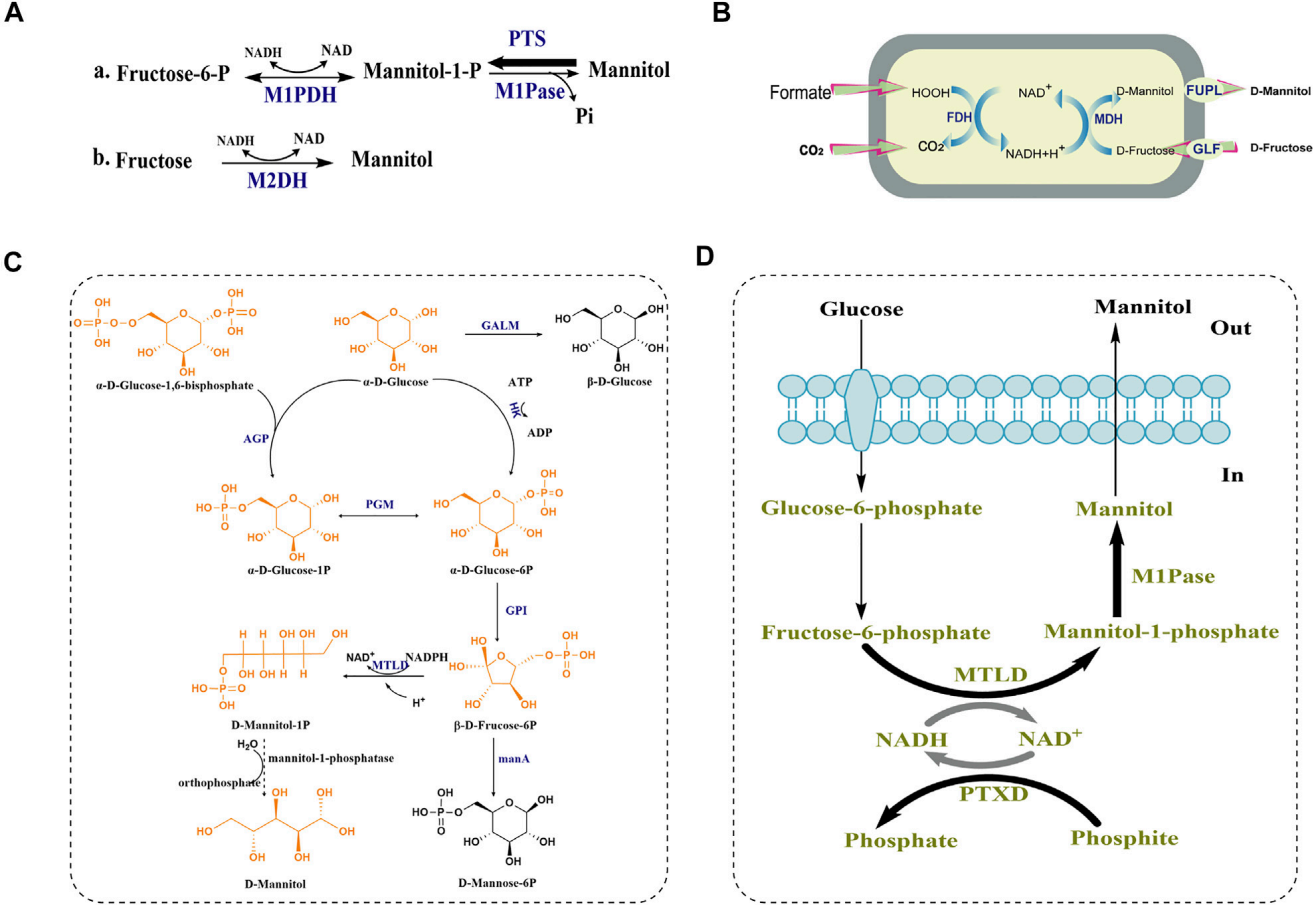
D-mannitol biosynthetic pathway in lactic-acid bacteria, *H. sinensis* and *E. coli*. **(A)** a. Proposed mannitol metabolism in non-lactic-acid bacteria and homofermentative lactic-acid bacteria and b. heterofermentative lactic-acid bacteria PTS: phosphoenolpyruvate-dependent mannitol phosphotransferase system. The size of the arrow indicates the trend of reaction. **(B)** A recombinant oxidation/reduction cycle in Escherichia coli for D-mannitol formation. **(C)** The predicted biosynthetic pathway of D-mannitol in H. sinensis. AGP: glucose pyrophosphorylase; GALM: galactose mutarotase; HK: hexokinase; PGM: phosphoglucomutase; GPI: glucose phosphate isomerase; MTLD:mannitol-1-phosphate dehydrogenas; manA: hexokinase-like mRNA coding protein (manA1-manA5). **(D)** Synthetic pathway constructed in *Escherichia coli* leading to mannitol production from glucose. Bold arrows indicate overexpression.

Lactic acid bacteria (LAB) are known to be a type of bacteria that own the capability of efficiently converting sugars into D-mannitol. Based on the latest report, fermentation of apple juice with the well-known D-mannitol-producing LAB strain *Leuconostoc citreum* TR116, the bioreactor reduction of sugar was scaled up to 98.6 g/L (83%) and the production of D-mannitol was achieved to 61.6 g/L (Rice et al., 2020). The high D-mannitol production of LAB made them into the first organism to be studied for D-mannitol biosynthesis. Thus, the biosynthesis of D-mannitol in LAB is the most clearly studied pathway at present (as shown in Figure 3A). In homofermentative lactic-acid bacteria, the synthetic D-mannitol is from fructose-6-P, under the catalysis of mannitol-1-phosphate dehydrogenase (M1PDH) and mannitol-1-phosphatase (M1Pase), and fructose-6-P is transformed into mannitol-1-P and then eventually form D-mannitol as shown Figure 3A a. However, the reaction that D-mannitol as a carbon source and converted into mannitol-1-P by a phosphoenolpyruvate-dependent specific phosphortransferase system (PTS) is the main reaction between mannitol-1-P and D-mannitol, and this pathway is usually used to degrade instead of form D-mannitol. Therefore, in order to establish efficient D-mannitol production, Xiao, H., *et al.* characterized how the D-mannitol genes (including M1PDH encoding gene *mtlD*, PTS encoding genes *mtlA* and *mtlF*, and the regulator MtlR encoding gene *mtlR*) in *L. lactis* were organized. Finally, by overexpressing the *mtlD* gene and using stationary-phase cells as biocatalysts, they attained 10.1 g/L D-mannitol with a 55% yield. To the best of our knowledge, this remains the highest titer ever reported for *L. lactis* (Xiao et al., 2021). By contrast, the D-mannitol from heterofermentative lactic-acid bacteria is the result of uptaking and utilizing fructose and D-mannitol is synthesized directly from fructose by mannitol-2-dehydrogenase (M2DH) without the synthesis of mannitol-1-P (Figure 3A,B) (Wei et al., 2021).

### Synthesis of D-Mannitol in Filamentous Fungi

Among filamentous fungi, *H. sinensis* is known to produce D-mannitol. In 2016, hexokinase and glucose phosphate isomerase were found to be involved in biosynthetic pathway of D-mannitol, due to their significant upregulation by 5.27-, and 3.94-fold according to real-time PCR. Moreover, judging from the glycolytic pathway and fructose-mannose pathway, Shan Lin *et al.* proposed a possible biosynthetic pathway of D-mannitol (shown in Figure 3C)*.*

In this pathway, α-D-glucose-1, 6-bisphosphate and α-D-glucose are converted by glucose pyrophosphorylase (AGP) into α-D-glucose-1P, which can be interconverted with α-D-glucose-6P by phosphoglucomutase (PGM). α-D-glucose is converted into α-D-glucose-6P under the catalysis of hexokinase (HK), and glucose phosphate isomerase (GPI) converts α-D-glucose-6P into β-D-fruccose-6P and the mannitol-1-phosphate dehydrogenase (MTLD) converts β-D-fruccose-6P into D-mannitol-1P (Lin et al., 2016b). However, the phosphatase that converts D-mannitol-1P into D-mannitol was not proposed, which might indicate that this enzyme in *H. sinensis* be un-annotated in the protein databases. There are two conceivable reasons to explain this finding. One is that the homology of mannitol-1-phosphatase in *H. sinensis* compared with the known mannitol-1-phosphatases from other species is low, and the other one is that certain unknown phosphatases may replace D-mannitol-1P for generating D-mannitol in *H. sinensis*. Additionally, there might be two branching reactions in this pathway that are not conducive to the synthesis of D-mannitol shown in Figure 3C. Firstly, the α-D-glucose at the beginning of the reaction can be converted into β-D-glucose under the catalysis of galactose mutarotase (GALM). Secondly, before D-mannitol-1P is produced, a part of the substrate β-D-fruccose-6P can be converted into D-mannose-6P under the action of a hexokinase-like mRNA coding protein (manA).

### Synthesis of D-Mannitol in Recombinant *Escherichia Coli*

D-mannitol can also be produced by constructing engineered *Escherichia coli*, which can be divided into the following three stages according to different substrate sources. Firstly, in 2004, a recombinant *E. coli* strain was constructed as biocatalyst for the whole-cell biotransformation of D-fructose into D-mannitol, and the introduction of a gene encoding a D-fructose transporter protein (GLF) from *Zymomonas mobilis* enabled the cells to import D-fructose (Figure 3B). Finally, the recombinant *E. coli* BL21 (DE3) expressing *glf*, *fdh* and *mdh* was able to form 362 mM D-mannitol from an initial 500 mM D-fructose of within 8 h, and the molar yield *Y*_D-mannitol/D-fructose_ reached 84% and a specific productivity was more than 4 g D-mannitol g^−1^ (cell dry weight) h^−1^ (Kaup et al., 2004). In order to overcome the instability of the recombinant strain, which possibly caused by factors such as the internal pH change of the cells, loss of cofactor NAD, high formate concentrations and export of D-mannitol, the *fupL* gene was additionally expressed in biotransformation experiments, and the final D-mannitol productivity of the strain was enhanced by 20% (Heuser et al., 2009).

Secondly, in order to convert D-glucose to D-mannitol, an intracellular glucose isomerase, formate dehydrogenase, D-mannitol dehydrogenase, glucose isomerase, and glucose facilitator were co-expressed in the *E. coli* strain I, resulting in the production of up to 821 mM D-mannitol at the optimized pH and temperature (Kaup et al., 2005). To construct a recombinant *E. coli* strain which can convert glucose into D-mannitol efficiently without the assistance of extracellular enzymes, Reshamwala et al. (2014) assembled a synthetic pathway (shown in Figure 3D) in *E. coli* to change carbon flow towards D-mannitol production by expressing two native enzymes from the protozoan parasite *Eimeria tenella*. Mannitol-1-phosphate dehydrogenase (MTLD) reduced fructose-6-phosphate to mannitol-1-phosphate, and mannitol-1-phosphatase (M1Pase) dephosphorylated mannitol-1-phosphate to yield D-mannitol. The reduction of fructose-6-phosphate is accompanied with the oxidation of NADH, and for the sake of regenerating NADH, the phosphite dehydrogenase (PTXD) of *Pseudomonas stutzeri* was expressed to make it the ability to catalyze the almost nonreversible oxidation of phosphite into phosphate, and at the same time NAD^+^ was reduced into NADH. D-mannitol was then transported across the membrane and released into the culture supernatant, whereby a molar yield of 87% was achieved without using complex media components and elaborate process control mechanisms (Reshamwala et al., 2014).

In recent years, new insights have emerged on the D-mannitol biosynthetic pathway, especially after the first algal genes involved in D-mannitol production were identified in the model brown alga *Ectocarpus* (Rathor et al., 2020). However, to accomplish simplifying the engineering process and generate ready-made protein modules, their functionality in heterologous systems remains to be tested. Madsen et al. (2018) expressed a mannitol-1-phosphate dehydrogenase (M1PDH) and a mannitol-1-phosphatase (M1Pase) by fusing an enzyme from the marine alga *Micromonas pusilla* in *E. coli* and successfully constructed simpler way to assemble and optimize recombinant metabolic pathways to produce the building blocks of D-mannitol.

To sum up, these three synthesis methods are currently different in the degree of industrial application. For LAB, it is an important group of microorganisms for fermentation of a large range of products, such as yogurt, soy sauce, antiseptic substance and so on. Its fermentation technology is relatively mature and controllable, so it is usually used in industry to produce D-mannitol by fermentation with fructose as a substrate. However, lactic acid bacteria fermentation requires a large amount of fructose, which is costly, and undesirable metabolites are difficult to remove. For filamentous fungi, D-mannitol is one of important active ingredients formed by direct reduction of fructose-6-phosphate, but the pathway between D-mannitol-1P and D-mannitol needs to be further verified. Once this approach is verified, we can try to apply it to the construction of mannitol cell factories. For recombinant *E. coli*, ⅰ) by introducing exogenous genes, substrates and product transporters, *E. coli* can be used as a biocatalyst to produce D-mannitol with fructose or glucose. ⅱ) by transferring exogenous fusion proteins into *E. coli* to produce D-mannitol.

## Cordyceps Polysaccharides

*C. militaris* polysaccharides represent a class of biological macromolecules with diverse structures and extensive physicochemical properties. Many studies have shown that polysaccharides isolated from artificial cultured *C. militaris* have diverse pharmacological activities, including antitumor (Ukai et al., 1983; Kim et al., 2001), anti-inflammatory and immunoregulatory activities (Yu et al., 2004a). On account of the diversities in raw materials, extraction and purification methods, different cordyceps polysaccharides with diverse structures and bioactivities have been extracted and identified from *C. militari*s. To the aspect of its clinical application, the main hindrance was the repeatability, reliability and consistency of cordyceps polysaccharides preparation (Zhang et al., 2019). Recently, scientists mainly put the focus on establishing a standard method for the extraction and preparation of cordyceps polysaccharides, the pharmaceutical activity of the obtained cordyceps polysaccharides and the study of the cordyceps polysaccharides biosynthesis pathway.

### Extraction, Purification and Identification of Cordyceps Polysaccharides

**FIGURE 4:**
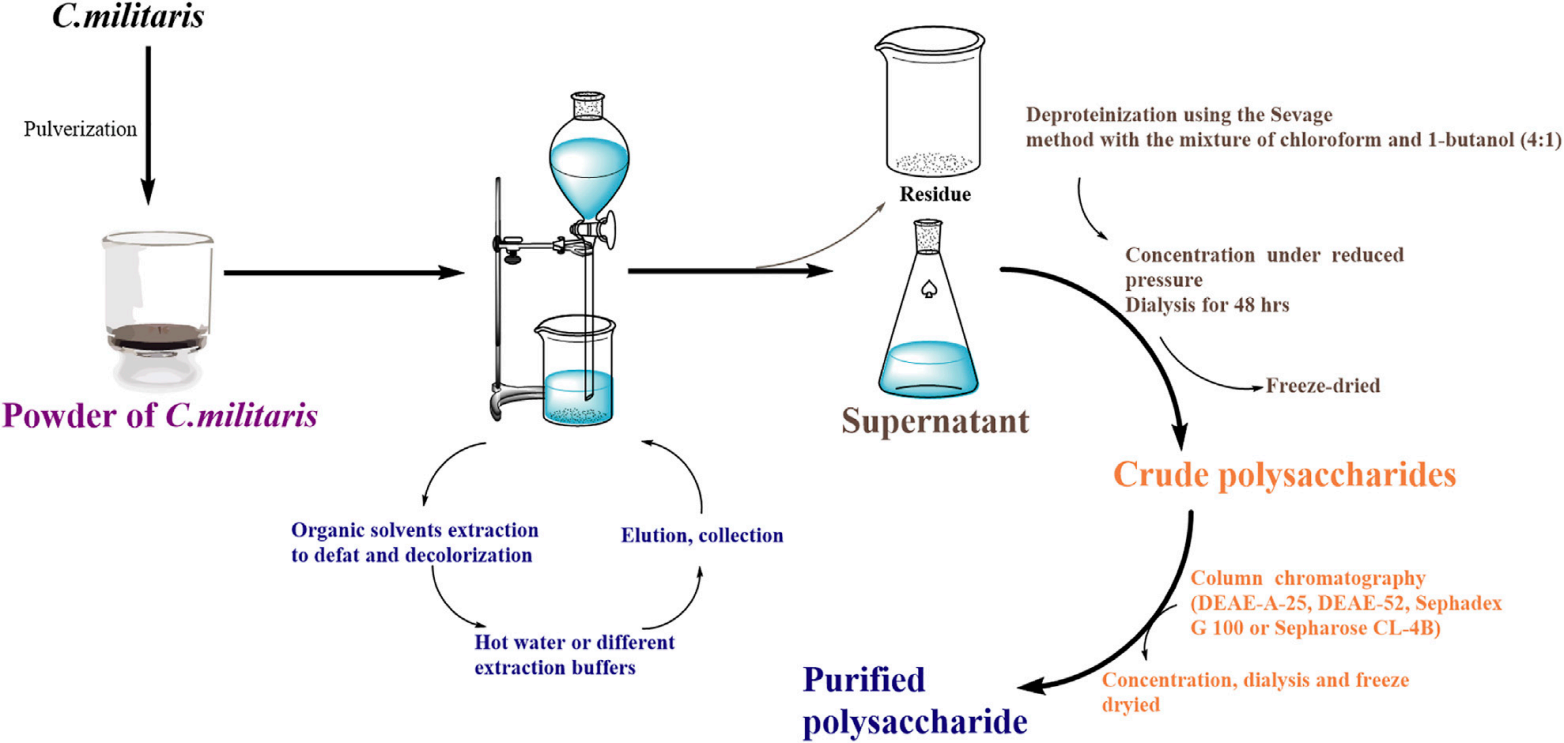
Flow chart of the purification of polysaccharides from *C. militaris*.

To our knowledge, many methods were used to extract polysaccharides from *C. militaris,* such as pure water, acidic/alkaline solutions, or heated buffer solutions as shown in Figure 4. Although hot or boiling water is the most convenient solvent for the extraction of cordyceps polysaccharides, the extraction time is too long and the extraction rate is too low, so some new extraction methods have recently been exploited to improve the extraction efficiency, such as microwave extraction, ultra-high pressure extraction, subcritical water extraction, ultrasonic extraction, and enzyme-assisted extraction (Zhang et al., 2019; Park and Lee, 2021), and the differences between these extraction methods are shown in Table 1.

**TABLE 1:** Comparison of extraction methods for polysaccharides.

Extraction methods	Advantages	Disadvantages
Hot or boiling water	Simple operation, wide range of applications, cheap solvent	Short storage time and many product impurities
Backflow method	Controllable temperature, less solvent consumption, simple operation	Low efficiency, long time
Enzyme-assisted extraction	Specificity, high efficiency and mild reaction conditions	Strict reaction conditions, the enzyme is easy to be inactivate, and the conditions need to be optimized
Ultrasonic extraction	Low extraction temperature, simple operation	Restricted by ultrasonic attenuation factors, the extraction rate is limited
Microwave extraction	High selectivity, simple and fast, can assist other extraction techniques to work together, save solvent, high extraction rate, low cost	Less samples processed at one time
Ultra-high pressure extraction	Preserve the activity of the extract	High equipment investment
Subcritical water extraction	Simple equipment, short extraction time, large selection of solvents, less pollution, wide application prospects	Use more solvents, longer extraction time, and relatively high cost

Due to the complex chemical structure of fungal polysaccharides, its extraction from the same raw material can also have different structures and exhibit different biological activities. In order to study the structures of these cordyceps polysaccharides in detail, many studies were done on the structure and function of cordyceps polysaccharides. At the beginning, most of the reports mainly focused on the extraction of cordyceps polysaccharides but didn’t further disclose the components, linkages and bioactivities of the extracted cordyceps polysaccharides due to the lack of valid methods. For example, as shown in Table 2, Kim S W *et al.* firstly obtained four groups of polysaccharides (named as Fr-I, Fr-II, Fr-III, and Fr-IV) from the culture filtrates of *C. militaris*, however, those components, linkages and bioactivities were not analyzed in detail (Kim, 2003).

**TABLE 2:** Polysaccharides from *C. militaris*: extraction methods, characteristics, chemical structures and bioactivities.

Living strains	Extraction	Category	Components	Linkages	Bioactivities	References
*Cordyceps militaris* NG3	size exclusion chromatography (SEC)	Fr-I	—	—	—	Kim, (2003)
		Fr-II				
		Fr-III				
		Fr-IV				
*Cordyceps militaris*	ethanol precipitation, deproteination and gel-filtration chromatography	CPS-2	Rha:Glc:Gla = 1: 4.46: 2.43	1→4, 1→6 linkages	—	Yu et al. (2004a); Yu et al. (2004b)
		CPS-3	D-glucose,ɑ-D-glucose	1→4, 1→6 linkages	—	
*Cordyceps militaris* Grown on Germinatd Soybeans	boiling water	APS	D-galactose,L-arabinose,D-xylose, L-rhamnose, and D-galacturonic acid	Araf-(1f, f5)-Araf-(1f, f4)-Galp-(1f and f4)-GalAp-(1f residues	modulation of the immune function of macrophages	Ohta et al. (2007)
*Cordyceps militaris*	hot water extraction and ethanol precipitation	CMP Fr I	—	—	—	Lee et al. (2010)
		CMP Fr II	Glu:Gal:Man = 3.28:1.53:1	1→2, 1→4, 1→6 linkages	Upregulate the phenotypic functions of macrophages	
		CMP Fr III	—	—	—	
*Cordyceps militaris*	hot water and precipitated by 50% ethanol	W-CBP50-II	ɑ-glucose,ɑ-mannose	ɑ-type glycosidic linkages	antioxidant activities	Chen et al. (2013)
			ɑ-galactose and ɑ-arabinose uronic acid and protein			
*Cordyceps militaris*	Soxhlet extraction using temperature gradient	β-(1→3)-D-glucan	D-glucan	1→3 linkages	anti-inflammatory	Smiderle et al. (2014)
*Cordyceps militaris*	DEAE-52 cellulose anion exchange column and a Sepharose	CMN1	L-rhamnose, L-arabinose, D-mannose, D-galactose	1→2, 1→3, 1→4, 1→6 linkages	anti-hypoxic	Dong et al. (2015)
	G-100 column					
*Cordyceps militaris*	Subcritical water extraction (SWE)	CMP-W1	Glu:Gal:Man = 1:1.29:2.84	—	immunostimulatory activity	Luo et al. (2017)
		CMP-S1	Glu:Gal:Man = 1:1.09:2.05	—		
*Cordyceps militaris*	Column chromatography	CM3-SⅡ	Glu:Gal:Man = 1:3.7:10.6	1→2, 1→4, 1→6 linkages	Hypolipidemic	Wang et al. (2021)
*Cordyceps militaris* cultivated on hull-less barley	hot water extraction and ethanol precipitation	SDQCP-1	Glu:Gal:Man = 1:9.7:13.3	1→2, 1→4, 1→6 linkages	antioxidant and immunomodulatory	Zhang et al. (2020c)

In 2004, ethanol precipitation, deproteination and gel-filtration chromatography were sequently applied to purify polysaccharides from the hot-water extracted *C*. *militaris*, and obtained four fractions as shown in Table 2. Their formula weights were detected using gel-filtration chromatography, and the structures of CPS-2 and CPS-3 were linked by 1→4 or 1→6 linkage, which were elucidated by sugar analysis, Smith degradation, IR and 13C–NMR spectroscopy (Yu et al., 2004b). In 2010, Lee J S *et al.* used DEAE-cellulose and Sepharose CL-6B column chromatography to obtain the crude water-soluble polysaccharide CMP (*C*. *militaris* polysaccharide) from the fruiting bodies of *C. militaris* after hot water extraction and ethanol precipitation, termed CMP Fr I, CMP Fr II, and CMP Fr III (Lee et al., 2010). Adequate evidences showed that these *C. militaris* polysaccharides own the activities of antioxidant (Chen et al., 2013), antiangiogenetic (Zeng et al., 2014), anti-inflammatory (Smiderle et al., 2014), anti-hypoxic (Dong et al., 2015), and immunostimulatory activities (Luo et al., 2017).

### The Biosynthesis of Cordyceps Polysaccharides

Researches on polysaccharides biosynthesis of other organisms, including overexpression of key polysaccharide biosynthetic genes (Zhou et al., 2018), joint expression of multiple genes (Qu et al., 2022), blocking polysaccharide synthesis bypass (Lei et al., 2020), etc. are all feasible ways to increase yield. However, because of the structural determination of cordyceps polysaccharides is still limited, the disclosure of synthesis pathway is not clear until now, the research level of polysaccharide biosynthesis is lower than that of other active substances in *Cordyceps*. This complicated situation has also led to the current lack of researches on the biosynthesis of cordyceps polysaccharides.

In 2016, a study by Shan Lin *et al.* (Lin et al., 2016a) opened a significant avenue for finding key enzymes involved in the cordyceps polysaccharide biosynthesis pathways. To enhance the cordyceps polysaccharide (CP) production from submerged cultivation of *H. sinensis*, 2 mM of manganese sulfate on day 0 was the optimal amount and timing of addition, and the CP production reached to the optimum with 5.33%, which was increased by 93.3% compared with the control. Notably, they found that the intracellular mannose content decreased by 43.1% during cultivation under manganese stimulation, and the consumption of mannose just corresponded with the accumulation of CP. This mannose biosynthesis pathway was the first verified pathway which constituted a vital section of CP biosynthesis, and the transcriptional levels of the biosynthetic genes indicated that the transcription of cordyceps polysaccharides synthesis related genes (*cpsA*) upregulated 5.35-fold significantly, implicating a crucial gene involved in both mannose and CP biosynthesis. However, the exact relationship between mannose and CP biosynthesis is still unknown.

### N^6^-(2-Hydroxyethyl)-Adenosine

N^6^-(2-hydroxyethyl)-adenosine (HEA) is a derivative of adenosine that was the first identified calcium ion channel antagonist from biological sources. As early as 1983, Furuya *et al.* isolated HEA from the hyphae of *Cordyceps* grown in liquid culture, and this pioneering finding was published in phytochemistry.

The molecular weight of HEA is 311.29, its molecular formula is C_12_H_17_N_5_O_5_, and its structural formula is shown in Figure 1E. It possesses many activities, such as protection against ultraviolet radiation, blood-thinning, anti-inflammatory (Li I.-C. et al., 2019; Wang X. et al., 2019; Chyau et al., 2021), analgesic, and antihypertensive activities (Li I.-C. et al., 2019), protective effects against diabetic kidney disease (Wang X. et al., 2019), as well as inhibiting the proliferation of tumor cells, protecting the brain (Zhang et al., 2016), and inducing apoptosis of gastric carcinoma cells (Xie et al., 2020). HEA is generally regarded as being at the intersection of medical care, cosmetics and biological defense. Consequently, it is considered the most important biologically active ingredient to measure the quality of *Cordyceps* products. Compared with other compounds obtained from *Cordyceps*, such as the well-known cordycepin, there are few patents related to HEA. The research on HEA is still in the early stage of exploration. At present, there are fewer studies on the screening and hybridization of HEA-producing strains, and more studies focused on the physiological effects of HEA.

**FIGURE 5:**
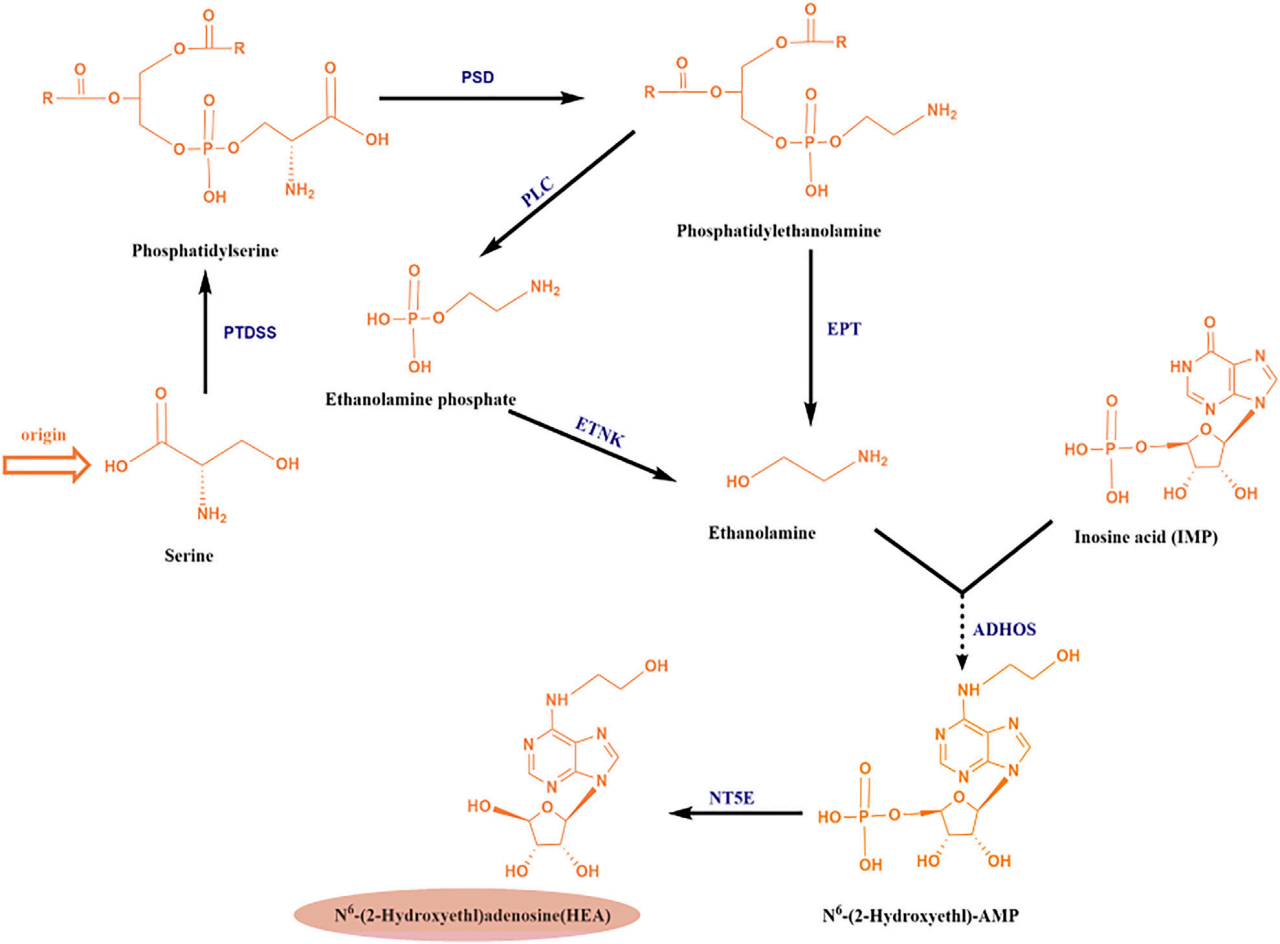
Analysis of the putative metabolic pathway of N^6^ -(2-hydroxyethyl) adenosine (HEA); Abbreviations: PTDSS, phosphatidylserine synthase; PSD, phosphatidylserine decarboxylase; EPT, ethanolamine-phosphotransferase; PLC, phospholipase C; ETNK: ethanolamine kinase; ADHOS, adenylohydroxyethyl synthetase; NT5E, 5′-nucleotidase.

Recently, an enzyme from *C. militaris* with 74% amino acid sequence similarity to adenylosuccinate synthetase (a key enzyme for adenosine synthesis) was identified by bioinformatic analyses, which suggested that the biosynthesis of HEA was probably similar to that of the conversion of inosine monophosphate (IMP) to adenosine (shown in Figure 2 in grey above). Accordingly, a hypothetic biosynthesis pathway for N^6^-(2-hydroxyethyl) adenosine in *C. militaris* was proposed as shown in Figure 5 (Chen et al., 2021). Similar to the conversion of IMP and L-aspartate into N^6^-(1, 2-dicarboxyethyl)-AMP by adenylosuccinate synthetase, IMP and ethanolamine can be converted into N^6^-(2-hydroxyethyl)-AMP by adenylyl hydroxyethyl synthetase, and N^6^-(2-hydroxyethyl)-AMP is subsequently dephosphorylated to yield HEA.

At present, there are no other report on the biosynthetic pathway of HEA, and in-depth study of its biosynthetic mechanism will open up new ways for the wide application of HEA. Therefore, elucidating the synthesis pathway of HEA at the protein level, identifying the HEA biosynthesis gene clusters, cloning the enzyme-coding genes related to HEA synthesis, and increasing the output of HEA through metabolic engineering, will be a breakthrough in the research of HEA.

## Concluding Remarks and Future Perspectives

*C. militaris* is closely related to the highly prized *O. sinensis*, both of them have been reported without toxicity based on the results of chromosomal aberration of CHL cells, Ames test, acute toxicity test and MN test of bone marrow cells. In addition, compared with *O. sinensis*, *C. militaris* has lower cost of cultivation (Gong et al., 2003; Gong et al., 2004; Long et al., 2021), which makes it a promising substitute for *O. sinensis*. In nature, the formation of *C. militaris* fruiting bodies requires a specific ecological environment and host insects, which leads to its scarcity as a natural resource. In view of the severe situation of the limited natural resources of *C. militaris* and the long artificial cultivation cycle, researches have attempted to increase the output of various bioactive components using strategies including chemical synthesis, microbial fermentation, and biosynthesis. And we compared these existing methods of obtaining the four active substances mentioned above in *Cordyceps* in Table 3. Among them, biosynthesis is undoubtedly the most popular and promising approach due to its low impact on the health and the environment.

**TABLE 3:** Comparison of four bioactives existing methods of acquisition.

Bioactives	Existing methods of acquisition	Productivity	Advantages	Disadvantages
Cordycepin	*C. militaris* fermentation	6,200 mg/L (fermentation broth) Sari et al. (2016)	Relatively high yield	Strains degeneracy, long fermentation cycle
		6.63 mg/g (sclerotium) Kang et al. (2017)		
	Chemical synthesis	36% (starting from adenosine) Huang et al. (2017)	Convenient, quick effect, controllable conditions	High cost for the treatment of the pollution
	Microbial cell factory	137.27 mg/L (Recombinant *Saccharomyces cerevisiae* fermentation broth) Huo et al. (2021)	Low cost and less pollution, short fermentation cycle, conducive to industrial production	Lower yield than *C. militaris* fermentation system
D-mannitol	Bioreactor fermentation	61.6 g/L (fermentation broth with fructose as substrate) Rice et al. (2020)	Relatively high yield	High fermentation cost, difficult to remove undesirable metabolites
	Chemical hydrgenation of high-fructose	65% (starting from D-sorbitol, D-glucose, D-fructose) Jin and Li (2006)	Relatively high yield	Requires high pressures, high temperatures, hydrogen gas, and raney nickel catalyst
	Biotransformation	87% (starting from glucose) Reshamwala et al. (2014)	High total sugar utilization, without the use of complex media components and elaborate process control mechanisms	Unstable over a long term
	Microbial cell factory	218 mg/L (fermentation broth) Madsen et al. (2018)	Short fermentation cycle, low cost and less pollution	Relatively low yield
Polysaccharides	*Cordyceps* fermentation	Varies due to different extraction and purification methods	—	Lack of standard method of polysaccharide collection
		Yield can be increased by homologous co-overexpression of genes involved in precursor nucleotide sugars biosynthesis Zhou et al. (2018)	Conducive to genetic manipulation and metabolic engineering to super-produce polysaccharides in other fungi	Need further research
N^6^-(2-hydroxyethyl) -adenosine	*Cordyceps* fermentation	94 mg/L (fermentation broth) Chunyu et al. (2019)	Relatively high yield	Strains degeneracy, long fermentation cycle
	Chemical synthesis	74% (acrylic acid and chloroethanol) Dong and Gao (1996)	High yield	High material cost and polluted

In future research, synthetic biology techniques can be used to verify the inferred cordycepin biosynthetic pathway. Metabolic engineering transformation, process engineering optimization and other strategies can be applied to optimize cell factories. For D-mannitol, optimize the cell factory in *E. coli* probably become the major trend of industrial application. Since the yield of D-mannitol obtained through these referred methods is relatively low, the specific control strategies need to be further studied. For instance, deleting pathways for carbon dissimilation that compete with D-mannitol biosynthesis, using optimized high cell density biotransformation and engineered strains to increase yield. Besides, we can also try to construct D-mannitol-producing strains with other strains with clear genetic background and simple operation. For cordyceps polysaccharides (CP), they have a variety of complex structures and compositions, and genes related to the production of CP are a large and diverse group, therefore, more in-depth researches are needed to explore CP synthesis and its regulatory mechanism. For HEA, its biosynthetic pathway has not been clearly reported so far, the speculated pathway mentioned in our review can be used as an entrance for studying HEA synthesis. In conclusion, this review provides evidence to further improve and study the biosynthesis of the bioactive components of *Cordyceps*, including cordycepin, D-mannitol, cordyceps polysaccharides and HEA.
